# Comparison of Sublingual and Intramuscular Vitamin B12 in Children With Nutritional Vitamin B12 Deficiency Megaloblastic Anemia

**DOI:** 10.7759/cureus.100895

**Published:** 2026-01-06

**Authors:** Nongthombam N Devi, Utkarsh Bansal, Nivedita P Yerramilli, Girjesh K Singh, Amit K Rastogi, Vinaya A Singh

**Affiliations:** 1 Pediatrics, Hind Institute of Medical Sciences, Barabanki, IND; 2 Pediatrics, Ramansh Child Care, Lucknow, IND

**Keywords:** hemoglobin (hb), hyperpigmented knuckles, hypersegmented neutrophils, macrocytic anemia, mean corpuscular volume (mcv) level, methylcobalamin, pancytopenia, parenteral, pediatric hematology, sublingual therapy

## Abstract

Background and aim

Vitamin B12 deficiency remains a major cause of nutritional anemia in children, particularly in low- and middle-income countries. While intramuscular (IM) vitamin B12 is the traditional treatment, the sublingual route offers a simpler, painless alternative with potentially comparable efficacy. Thus, this study was planned to compare the efficacy of sublingual versus IM vitamin B12 supplementation in children with confirmed vitamin B12 deficiency anemia.

Materials and methods

A prospective comparative study was conducted among children aged 1-15 years at a tertiary care center in Uttar Pradesh. Participants were randomized by using a computer-generated table into two groups: Group A received IM vitamin B12, and Group B received sublingual vitamin B12 for 12 weeks. Blinding of study subjects or the investigating officer was not possible due to obvious differences in the routes of administration of the drugs (IM vs. sublingual). However, the outcome assessors, including the laboratory personnel and the statistician, were blinded to avoid bias. The allocation concealment was performed using the sequentially numbered, opaque, sealed envelopes (SNOSE) method to minimize selection bias by the investigating officer. Hematological parameters, such as hemoglobin (Hb), mean corpuscular volume, total leucocyte count, platelet count, and serum B12, were evaluated at baseline, one month, and three months. Our primary outcome measures were correction of anemia beyond WHO age-specific thresholds and correction of B12 levels to >300 pg/mL as per the Indian Academy of Pediatrics guidelines.

Results

A total of 73 patients (Group A: 36 and Group B: 37) with comparable baseline clinical and hematological characteristics participated in the study. The baseline mean ± SD Hb levels were 8.33 ± 1.14 g/dL in Group A and 8.46 ± 1.24 g/dL in Group B, while the mean ± SD B12 levels were 132.53 ± 38.33 pg/mL and 141.20 ± 31.65 pg/mL in Groups A and B, respectively. The mean ± SD Hb levels at three months were 12.07 ± 0.78 g/dL and 12.31 ± 0.73 g/dL in Group A and Group B, respectively, showing a significant increase from baseline, although the results were comparable across both groups. Statistically significant increments of serum B12 levels (Group A: 329.30 ± 34.94 pg/mL; Group B: 337.08 ± 44.19 pg/mL) were also noted. Normalization of B12 levels was achieved in 100%, while a nonanemic status was achieved in 80.8% (59/73) of the participants. Both routes were well tolerated, and no adverse events were noted during the study period.

Conclusions

Sublingual vitamin B12 is as effective as IM administration for pediatric B12 deficiency anemia. Although the parenteral route remains the gold standard in cases with neurological involvement or severe thrombocytopenia, the sublingual route may be considered for maintenance therapy, as it is a noninvasive, child-friendly alternative that also offers a distinct cost benefit. Therefore, the sublingual route may be considered a potential option, pending evidence from larger, multicenter trials with longer follow-up periods.

## Introduction

Anemia is defined by WHO [1] as a hemoglobin (Hb) concentration below 2 SDs from the population mean. In low- and middle-income countries like India, nutritional deficiencies remain a major cause of this condition. According to the National Family Health Survey (NFHS-5), the highest prevalence of anemia was noted to be among children <5 years of age, with iron deficiency being the most common cause. This was followed by B12 and folate deficiency, accounting for nearly one-third of anemias in this age group. The prevalence of B12 and folate deficiency was found to be higher in school-going and adolescent age groups. In about 30% of the cases, a combined deficiency may also exist [2].

In a retrospective analysis conducted in North India, the prevalence of B12 deficiency was found to be as high as 47% among the general population [3]. Vitamin B12 is particularly deficient in plant-based sources, explaining the higher prevalence of deficiency among the strictly vegetarian and vegan population of our country [4].

Megaloblastic anemia was earlier treated predominantly by the intramuscular (IM) route of B12 due to a presumed poor intestinal absorption. However, in order to correct the B12 stores, the ideal duration of treatment may extend up to as long as six months. Administration of repeated IM injections is not a convenient option, especially in the pediatric population, due to the associated pain, discomfort, and need for expertise [5].

According to literature, in the adult population, the oral route is as effective as the parenteral route and is more often preferred due to ease of administration [6]. However, in children belonging to low- and middle-income countries, due to the high prevalence of gastrointestinal infections and infestations, absorption through the oral route may be unreliable.

In recent studies, the sublingual route of B12 has been gaining popularity even in those with associated malabsorption. When given through the sublingual route, vitamin B12 gets directly absorbed into the capillary circulation, thereby bypassing the intrinsic factor-dependent absorption from the terminal ileum [7].

There are limited studies comparing sublingual and IM routes of B12 in the pediatric age group. As the current evidence in this area is limited to a few single-arm studies [5] or randomized trials [8] with small sample sizes, we conducted a study to compare the efficacy of the sublingual route with that of the standard IM route in children with established vitamin B12 deficiency anemia.

## Materials and methods

This prospective comparative analytical study was conducted at a tertiary care center in the Barabanki district of central Uttar Pradesh over a period of 18 months from January 2024 to June 2025. The primary objective was to compare the efficacy of sublingual and IM B12 with respect to improvement in vitamin B12 levels in patients with proven B12 deficiency anemia. Our secondary objectives were to compare the improvements in parameters such as Hb, mean corpuscular volume (MCV), total leukocyte count (TLC), and platelet counts. The primary outcome measures were correction of anemia in the form of improvement of Hb levels beyond WHO age-specific thresholds [1] and correction of B12 levels to >300 pg/mL as per the Indian Academy of Pediatrics guidelines [2].

In a study conducted in an adult population, the SD of vitamin B12 after four months of therapy was 165 pg/mL and 595 pg/mL in the IM and oral groups, respectively, and the reported B12 levels were 306 pg/mL and 643 pg/mL, having a difference of 337 pg/mL. Assuming the minimum expected mean difference of vitamin B12 levels between the groups to be 300 pg/mL, a 5% level of significance, a power of 80%, and a dropout rate of 10%, the sample size was calculated as 35 in each group [9].

All children between 1 and 15 years of age presenting to the department of pediatrics who had clinical signs and/or symptoms of anemia (irritability, weakness, easy fatiguability, dyspnea on exertion, and pallor) or vitamin B12 deficiency (icterus, glossitis, knuckle hyperpigmentation, hepatomegaly, and splenomegaly) were enrolled. Also, children who were incidentally diagnosed to have Hb levels below WHO cutoffs (Table 1) for anemia were enrolled in the study [1].

**Table 1:** WHO anemia classification Hb, hemoglobin Source: World Health Organization (2024) [1]; Creative Commons Attribution (CC BY) license

Age group	Anemia if Hb (g/dL)	Severity (individual)
6-23 months	<10.5	Mild: 9.5-10.4
		Moderate: 7.0-9.4
		Severe: <7.0
24-59 months	<11.0	Mild: 10.0-10.9
		Moderate: 7.0-9.9
		Severe: <7.0
5-11 years	<11.5	Mild: 11.0-11.4
		Moderate: 8.0-10.9
		Severe: <8.0
12-14 years (boys), ≥12 years (girls: nonpregnant)	<12.0	Mild: 11.0-11.9
		Moderate: 8.0-10.9
		Severe: <8.0
≥15 years (boys)	<13.0	Mild: 11.0-12.9
		Moderate: 8.0-10.9
		Severe: <8.0

CBC, general blood picture (GBP), and reticulocyte counts were sent in each of these cases, and additional investigations were sent on a case-by-case basis. Vitamin B12 levels were sent for all children with anemia and the presence of any of the following features: elevated MCV (>100 fL), macrocytes or macro-ovalocytes, thrombocytopenia (platelet count <1.5 lakh/mm³), hyper-segmented neutrophils (>5 lobes in at least 5% of neutrophils or a single neutrophil with 6 or more lobes), pancytopenia [9] (Hb <10 g/dL, TLC <4,000/mm³, and platelet count <100,000/mm³), and decreased reticulocyte count (normal 0.5-1.5% of total red cell counts) [10]. Vitamin B12 levels were analyzed by using the Maglumi 800 chemiluminescence immunoassay analyzer (Snibe Diagnostic, Shenzhen, China).

All those with documented anemia and B12 levels <200 pg/mL [2] were considered for inclusion in our study. Children with borderline values of B12 (200-300 pg/dL) were not included, as they needed further evaluation with the use of functional markers like methylmalonic acid and homocysteine levels in order to prove that their anemia was secondary to B12 deficiency. Patients with clinical features or CBC and/or GBP findings suggestive of co-existing iron deficiency were also evaluated by sending an iron profile with serum ferritin levels.

Children with B12 deficiency associated with neurological involvement and/or severe thrombocytopenia (platelet count <50,000/mm³) were excluded from the study, as both are absolute indications for parenteral B12 administration [2]. Children <1 year of age were also excluded, as administration of the crushed sublingual tablet would be a practical concern. Those requiring packed red blood cell (PRBC) transfusions or those who had received PRBCs within the past month were excluded from the study. Children who received multivitamin supplements within one month of presentation were also excluded from the study.

A detailed history, including demographic data and dietary history, was noted. A thorough anthropometry, general, and systemic examination was done for all study subjects. The groups were allocated by randomization using a computer-generated randomization table. Blinding of study subjects or the investigating officer was not possible due to obvious differences in the routes of administration of the drugs (IM vs. sublingual). However, the outcome assessors, including the laboratory personnel and the statistician, were blinded to avoid bias. The allocation concealment was performed using the sequentially numbered, opaque, sealed envelopes (SNOSE) method to minimize selection bias by the investigating officer.

Group A received injectable vitamin B12 (Injection HCM 1000 µg/mL, Nutrigold India Pvt Ltd., Mumbai, India) at a dose of 1000 µg intramuscularly every alternate day for one week, followed by weekly for three weeks, and monthly for two months. Group B received sublingual vitamin B12 (Micob SL 1500 µg, Nutrigold India Pvt Ltd.) at a dose of 1500 µg daily for one week, every other day for two weeks, twice a week for three weeks, and once a week for six weeks [11]. The parents were trained to crush the tablet and place it in the oral cavity below the tongue in children <3 or those who were noncooperative for sublingual intake of the drug. Following this, the investigating officer, a team including postgraduate resident doctors and interns, communicated with the patients telephonically via audio or video calls, offering timely reminders and thereby ensuring proper technique. We also objectively checked the compliance by asking the parents to bring the empty blister packs of the sublingual tablets on the subsequent visits.

Those with combined B12 and iron deficiency were additionally given oral conventional iron at a dose of 2-3 mg/kg/day. All patients received oral folic acid supplementation at a dose of 1-5 mg/day as per recommendation [2]. The parents of the children in both groups were given age-appropriate dietary and nutritional advice by a trained dietician.

The children in both groups were followed up one week after initiation of therapy to look for clinical improvement. After four weeks of B12 therapy, Hb levels and reticulocyte counts were documented, and after 12 weeks, Hb, TLC, platelet counts, MCV, and B12 levels were repeated. At all visits, patients were asked about any adverse effects and tolerability.

The study was commenced after prior approval from the Institutional Ethics Committee. Informed consent was taken from the parents/guardians of all participating children; additionally, assent was taken where applicable.

IBM SPSS Statistics for Windows, Version 24.0 (Released 2016; IBM Corp., Armonk, NY, USA) was used to analyze descriptive variables. A per-protocol analysis was conducted, including only participants who completed the assigned intervention and the three-month follow-up assessment. Participants with missing follow-up data were excluded from the final analysis, and no imputation of missing data was performed. Efficacy endpoints were analyzed using an intention-to-treat (ITT) approach, including all randomized participants. A Linear Mixed Model (LMM) was employed with “group,” “time,” and “group-by-time interaction” as fixed effects and “subject” as a random effect. This model accounts for missing data under the missing-at-random assumption. Quantitative data were displayed as the mean and SD, while categorical variables were represented as percentages. The chi-squared test was employed for categorical data, and the independent t-test for numerical data when comparing two groups. The nonparametric data were subjected to the Mann-Whitney U test. p-Values below 0.05 were regarded as statistically significant.

## Results

**Figure 1:**
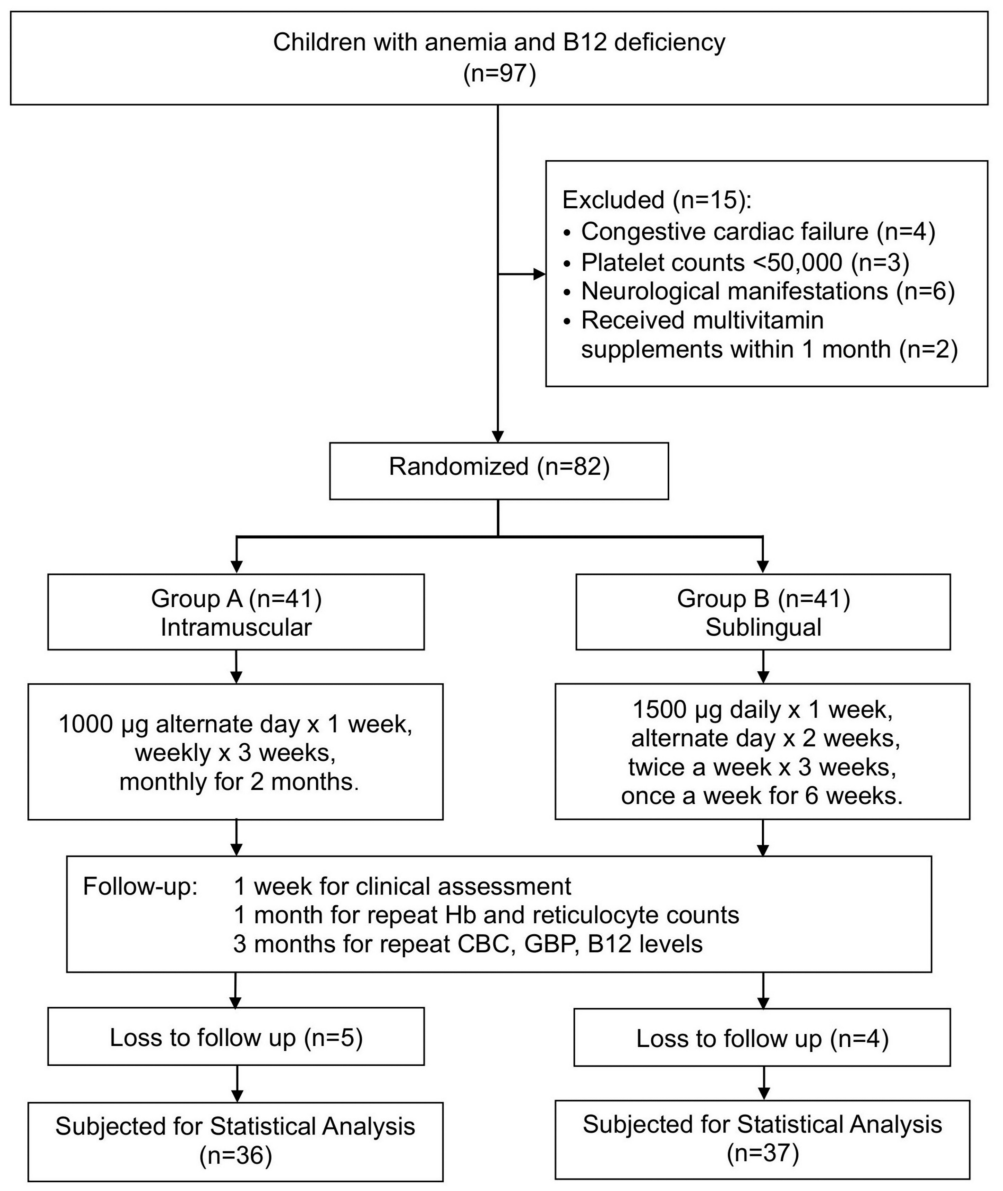
Flow of recruitment of participants in the study GBP, general blood picture; Hb, hemoglobin; IM, intramuscular

During the study period, a total of 97 patients with laboratory-proven vitamin B12 deficiency anemia were enrolled. After applying the inclusion and exclusion criteria, 82 children were randomized into Group A and Group B to receive IM and sublingual B12, respectively. However, nine patients were lost to follow-up. Finally, 73 children participated in the study, with Group A having 36 (50.7%) and Group B having 37 (49.3%) patients. Figure 1 depicts the flow of recruitment of participants.

Both groups had comparable baseline characteristics like age, gender, and clinical characteristics. The preliminary investigations, like CBC, GBP, reticulocyte counts, and vitamin B12 levels, were also comparable between the two groups. The prevalence of associated iron deficiency anemia was also equally distributed in both groups. Table 2 shows the baseline characteristics of the study participants. The Shapiro-Wilk normality test results showed that the baseline vitamin B12 values are normally distributed (p = 0.22).

**Table 2:** Comparison of baseline parameters between the two study groups Data are presented as n (%) or mean (SD).

Baseline characteristics	Group A (n = 37)	Group B (n = 36)	Statistical test value	p-Value
Age (years)				
1-5	7 (18.9)	6 (16.7)	ꭓ² = 1.1746	0.556
6-11	20 (54.1)	16 (44.4)		
12-15	10 (27)	14 (38.9)		
Mean age ± SD (years) (range)	8.53 ± 3.68 (1.8-15.0)	9.48 ± 3.62 (3-15.0)	t = 1.051	0.297
Male	16 (43.2)	21 (58.3)	ꭓ² = 1.6395	0.2004
Vegetarian	28 (75.7)	30 (83.3)	ꭓ² = 0.6464	0.4214
Weakness	3 (8.1)	2 (5.5)	ꭓ² = 0.1838	0.6681
Irritability	4 (10.8)	4 (11.1)	ꭓ² = 0.0017	0.9675
Easy fatigue	3 (8.1)	1 (2.77)	ꭓ² = 0.9873	0.3204
Dyspnea on exertion	1 (2.7)	1 (2.77)	ꭓ² = 0.0004	0.9844
Pallor	14 (37.8)	14 (38.9)	ꭓ² = 0.0084	1
Icterus	1 (2.7)	1 (2.8)	ꭓ² = 0.0004	1
Glossitis	13 (35.1)	11 (30.6)	ꭓ² = 0.1710	0.6792
Knuckle pigmentation	27 (73)	22 (61.1)	ꭓ² = 0.8057	0.3694
Hepatomegaly	4 (10.8)	2 (5.6)	ꭓ² = 0.8371	0.3602
Splenomegaly	5 (13.5)	4 (11.1)	ꭓ² = 0.0961	0.7566
Iron deficiency	10 (27)	14 (38.9)	ꭓ² = 1.1475	0.281

Table 3 shows that no statistically significant differences were observed between Group A and Group B for any key baseline demographic or biochemical variable (all p > 0.05). The 95% CIs for mean differences included zero for all parameters, confirming that the groups were comparable at baseline.

**Table 3:** Comparison of key baseline characteristics of participants between the two study groups Data are presented as mean (SD). Hb, hemoglobin; MCV, mean corpuscular volume

Variable	Group A (n = 37)	Group B (n = 36)	Mean difference (95% CI)	t-Value	p-Value
Age (years)	8.53 (3.68)	9.48 (3.62)	-0.93 (-2.70 to 0.83)	1.051	0.297
Hb (g/dL)	8.33 (1.14)	8.46 (1.24)	-0.18 (-0.74 to 0.38)	0.467	0.642
MCV (fL)	94.7 (10.1)	93.8 (11.12)	0.88 (-4.13 to 5.88)	-0.362	0.718
Baseline vitamin B12 (pg/mL)	132.53 (38.33)	141.20 (31.65)	-8.62 (-25.29 to 8.05)	1.052	0.296

After one week of initiation of therapy, the patients were assessed for subjective improvement in symptoms by telephonic interviews. In Group A, the number of participants reporting symptoms of anemia decreased from 11 to 7 (18.9%), while in Group B, it declined from 8 to 5 (16.4%). This decline indicates an overall improvement in the symptom profile among participants in both groups following the intervention.

The median (IQR) Hb levels assessed after one month of initiation of therapy in Group A and Group B were 9.30 g/dL (8.50-9.95) and 9.40 g/dL (8.38-10.20), respectively. Although both groups individually had significant increments in Hb from baseline, both routes had comparable results.

The reticulocyte counts at one month after initiation of therapy showed a rising trend with a value of 1.2 ± 0.10% (baseline: 0.9 ± 0.11) in Group A and 1.19 ± 0.09% (baseline: 0.85 ± 0.1) in Group B, thereby ascertaining the marrow's response to B12 supplementation. There was no comparable difference in the percentage increment across both groups.

The median (IQR) Hb levels at three months in Group A and Group B were 12.0 g/dL (11.7-12.8) and 12.4 g/dL (11.93-12.98), respectively, with no significant difference. Although both routes were equally effective, 6/37 (16.2%) children in Group A and 8/36 (22.2%) children in Group B remained anemic. No comparable statistical significance was noted.

As demonstrated in Table 4, both treatment protocols demonstrated a substantial effect size (Cohen’s d > 3.0) in improving clinical parameters over three months. However, the inter-group analysis revealed small to negligible effect sizes (d < 0.35) at the study endpoint, suggesting that neither treatment was vastly superior to the other in terms of final clinical outcome.

**Table 4:** Inter-group and intra-group comprehensive effect size analysis ᴬ⁰ Mean (SD) values of Group A at baseline ᴬ³ Mean (SD) values of Group A at three months ᴮ⁰ Mean (SD) values of Group B at baseline ᴮ³ Mean (SD) values of Group B at three months Data are presented as mean (SD).

Characteristics	Time interval	Group I, mean (SD)	Group II, mean (SD)	Mean difference	Effect size (d)
Hb (g/dL)	Intergroup baseline (A vs. B)	8.33 (1.14)ᴬ⁰	8.46 (1.24)^ᴮ^^⁰^	0.13	0.11
	Intergroup three months (A vs. B)	12.07 (0.78)ᴬ³	12.31 (0.73)ᴮ³	0.24	0.32
	Intragroup A (baseline vs. three months)	8.33 (1.14)ᴬ⁰	12.07 (0.78)ᴬ³	3.74	3.83
	Intragroup B (baseline vs. three months)	8.46 (1.24)^ᴮ^^⁰^	12.31 (0.73)ᴮ³	3.85	3.78
Vitamin B12 levels (pg/mL)	Intergroup baseline (A vs. B)	132.53 (38.33)ᴬ⁰	141.20 (31.65)^ᴮ^^⁰^	8.67	0.25
	Intergroup three months (A vs. B)	329.3 (34.94)ᴬ³	337.08 (44.19)ᴮ³	7.78	0.2
	Intragroup A (baseline vs. three months)	132.53 (38.33)ᴬ⁰	329.3 (34.94)ᴬ³	196.77	5.37
	Intragroup B (baseline vs. three months)	141.20 (31.65)^ᴮ^^⁰^	337.08 (44.19)ᴮ³	195.88	5.1

After three months, we documented the MCV, TLC, and platelet counts in all patients. Serum B12 levels were repeated at the end of three months and were noted to fall within the normal range (Group A: 329.30 ± 34.94 pg/mL; Group B: 337.08 ± 44.19 pg/mL) in 100% of our study subjects, with no statistical difference across the groups. Table 5 summarizes the hematological parameters and B12 levels before and after completion of treatment in both groups.

**Table 5:** Comparison of hematological parameters and vitamin B12 level at baseline and at three months between the two study groups Data are presented as mean (SD). Hb, hemoglobin; MCV, mean corpuscular volume

Characteristics	Time interval	Group A (n = 37)	Group B (n = 36)	t-Value	p-Value
Hb (g/dL)	Zero month	8.33 (1.14)	8.46 (1.24)	0.467	0.6423
	Three months	12.07 (0.78)	12.31 (0.73)	1.356	0.1792
WBC (× 10³/mm³)	Zero month	7.23 (1.67)	6.6 (1.8)	-1.551	0.1254
	Three months	7.6 (1.4)	7.03 (1.3)	-1.801	0.0759
Platelet (× 10^⁶^/mm³)	Zero month	2.45 (0.92)	2.4 (0.1)	-0.324	0.7468
	Three months	2.56 (0.87)	2.5 (0.93)	-0.285	0.7767
Reticulocyte count (% of total red cell counts)	Zero month	0.9 (0.11)	0.85 (0.1)	-2.03	0.0461
	Three months	1.6 (0.24)	1.5 (0.23)	-1.818	0.0733
MCV (fL)	Zero month	94.7 (10.1)	93.8 (11.12)	-0.362	0.7183
	Three months	86.78 (6.09)	85.22 (4.82)	-1.211	0.229
Vitamin B12 levels (pg/mL)	Zero month	132.53 (38.33)	141.20 (31.65)	1.052	0.296
	Three months	329.3 (34.94)	337.08 (44.19)	0.836	0.406

**Figure 2:**
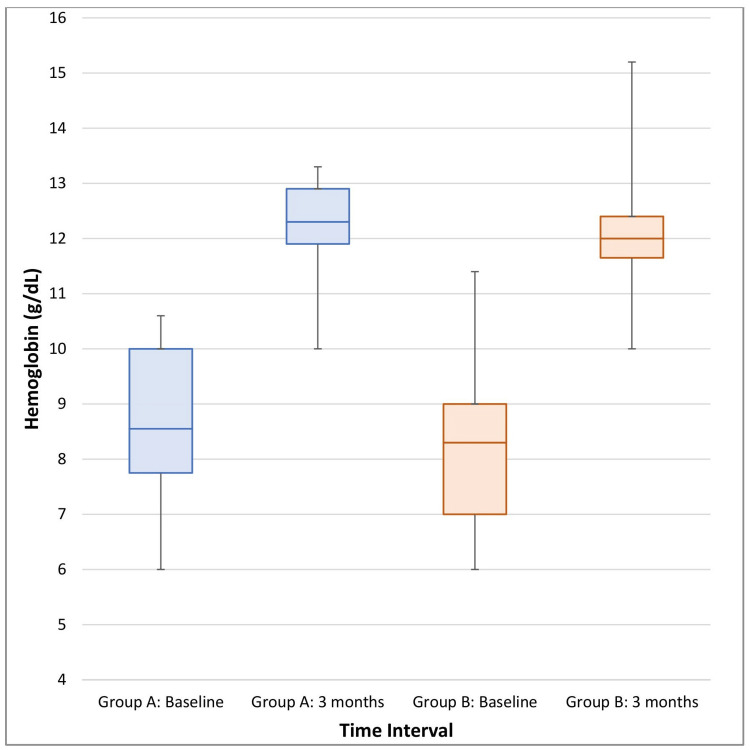
Box plot showing levels of Hb: baseline and after three months of treatment in both the study groups Hb, hemoglobin

The median (IQR) Hb levels at baseline in Group A and Group B were 8.30 g/dL (7.00-9.00) and 8.55 g/dL (7.25-9.83), respectively. After three months of therapy, the median (IQR) Hb levels in Group A and Group B increased to 12.0 g/dL (11.7-12.8) and 12.4 g/dL (11.93-12.98), respectively. Figure 2 depicts significant but comparable gains in Hb levels over three months after completion of therapy in both groups. This suggests both interventions were effective.

**Figure 3:**
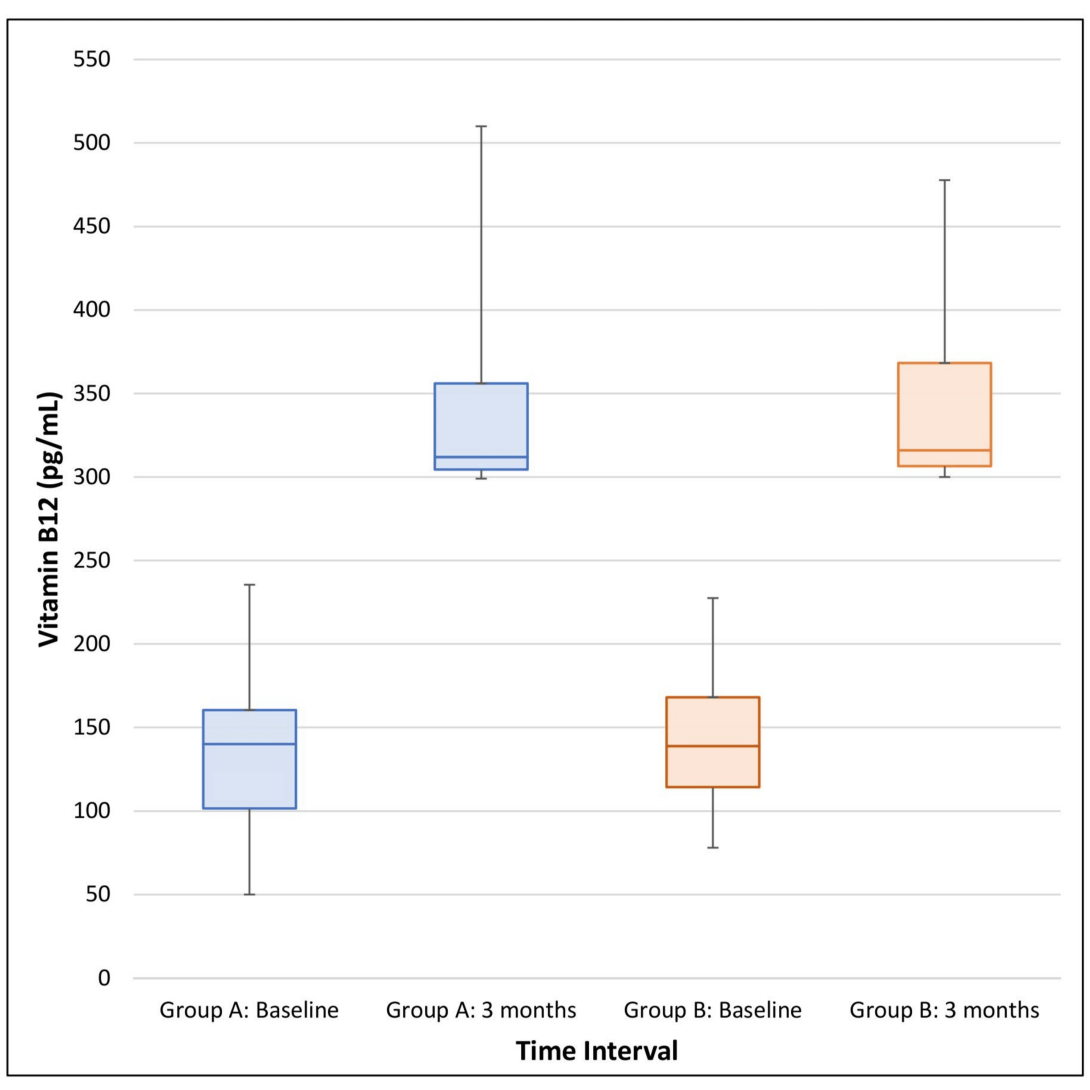
Box plot showing levels of vitamin B12: baseline and after three months of treatment in both the study groups

The median (IQR) serum vitamin B12 levels at baseline in Group A and Group B were 140 pg/mL (101.5-160.5) and 141 pg/mL (114.62-168.75), respectively. After three months of therapy, the median (IQR) B12 levels in Group A and Group B increased to 312 pg/mL (299-433) and 316 pg/mL (300-423), respectively. Figure 3 shows that both groups showed marked improvement in vitamin B12 levels over three months, with similar median values after intervention. The levels of all the participants were in the normal range post-therapy.

Analysis of the ITT population revealed significant improvements in both groups. The estimated marginal mean (EMM) for Hb at three months was 12.1 g/dL (95% CI: 11.8-12.4) in Group A and 12.3 g/dL (95% CI: 12.0-12.6) in Group B. The between-group difference was not statistically significant (mean difference: -0.2; p = 0.32).

We performed an ITT analysis using an LMM to account for missing data and repeated measures. The results for hematological and biochemical parameters are summarized in Table 6.

**Table 6:** ITT analysis of clinical parameters using LMMs Values are presented as EMMs with 95% CIs, derived from an LMM using restricted maximum likelihood estimation. EMM, estimated marginal mean; ITT, intention-to-treat; LMM, linear mixed model

Outcome variable	Time point	Group A (n = 37) (mean (95% CI))	Group B (n = 36) (mean (95% CI))	p-Value (time effect)	p-Value (interaction)
Hb (g/dL)	Baseline	8.33 (7.94-8.71)	8.50 (8.11-8.90)	<0.001	0.42
	Three months	12.01 (11.77-12.25)	12.34 (12.10-12.59)		
Vitamin B12 (pg/mL)	Baseline	132.53 (120.95-144.11)	140.41 (128.67-152.15)	<0.001	0.558
	Three months	332.92 (319.74-346.10)	333.94 (320.58-347.31)		

There was a highly significant main effect of time (F₁,₇₁ = 1491.4, p < 0.001), with both groups achieving normal or near-normal Hb levels by three months. Group A improved from a baseline mean of 8.33 g/dL (95% CI: 7.94-8.71) to 12.01 g/dL (95% CI: 11.77-12.25). Similarly, Group B improved from 8.50 g/dL (95% CI: 8.11-8.90) to 12.34 g/dL (95% CI: 12.10-12.59). The group-by-time interaction was not statistically significant (p = 0.420), indicating that the rate and magnitude of Hb recovery were comparable between the IM and sublingual routes.

Serum vitamin B12 levels showed a similar trajectory. A robust time effect was observed in both arms (F₁,₇₁ = 1142.2, p < 0.001). At three months, the EMM for vitamin B12 was 332.92 pg/mL in Group A and 333.94 pg/mL in Group B. The overlapping CIs and the nonsignificant interaction term (p = 0.558) confirm that both administration routes were equally effective in restoring serum vitamin B12 concentrations.

Apart from injection site-associated pain, both routes were equally tolerated. A few children complained of an aftertaste following the sublingual route. No adverse effects needing withdrawal of therapy were encountered.

## Discussion

This was a comparative analytical study wherein 73 children were randomized into two groups to receive IM or sublingual B12 therapy. Both Hb and B12 levels demonstrated a significant rise from the baseline in both groups, although the results across both groups were comparable, proving that the sublingual route was as effective as the IM route. A normal B12 value was achieved in 100% of the participants and a nonanemic status in 80.8% of the participants, thereby meeting the primary outcome measures.

As iron deficiency continues to rank as the topmost cause of nutritional anemia, we included it as an associated baseline character. We documented an iron profile whenever there was a clinical and/or hematological indication. The children receiving iron therapy constituted 32.8% (24 patients) of the participants, and these cases were equally distributed across the groups, similar to the Tandon et al. study [12].

We included children with B12 deficiency aged one year and above under the presumption that neurological manifestations were more common in infancy, and also because administration by the sublingual route would be challenging. In contrast to this general understanding, a study conducted by Varkal and Karabocuoglu [13] on the use of sublingual B12 in infants noted that they had a significant rise in B12 levels from the baseline.

The lack of a standardized dosing schedule was one of the major challenges faced while designing our methodology. In a similar study conducted by Tuğba-Kartal and Çağla-Mutlu [14], the authors used sublingual, oral, and parenteral routes at varying doses, frequencies, and durations. We designed a protocol similar to the one used by Orhan Kiliç et al. [8]. As there was limited literature on dosing, we used a readily available tablet with a strength of 1500 µg.

While administering the tablet, we exerted caution in children below 3 years of age in order to avoid accidental aspiration and choking. In order to overcome the risk, we ensured that the caregiver crushed the tablet before placing it sublingually. Also, we offered timely reminders telephonically in order to ensure compliance. Orhan Kiliç et al. [8] also conducted a similar study in children, where a B12 puff for sublingual delivery of the drug was used instead.

Bensky et al. [7] and Abdelwahab et al. [15] conducted studies comparing the efficacy of different routes of B12 therapy. Akin to our study, they also demonstrated an equal efficacy of the IM, oral, and sublingual routes in the improvement of B12 levels. Their studies highlight that the sublingual route should be equally considered in every patient with nutritional B12 deficiency.

Our study highlighted a distinct cost advantage of the sublingual route over the IM route. A total of 8 doses of injections (each ₹21) and 25 tablets (each ₹20.2) were needed during the three months. To seek treatment, the children in the IM group needed eight hospital visits, in contrast to two visits in the sublingual group. When an expected additional expense of approximately ₹260 was added per hospital visit [16], the total estimated expense was ₹2,088 in the IM group as opposed to ₹1,025 in the sublingual group for each patient.

Our meticulous telephonic reminder system, which ensured both patient compliance and care, was one of our biggest strengths. As our dosing schedule was complicated to start with, we designed a chart wherein the dates of the IM and sublingual doses were clearly written. The patients were also telephonically contacted to ensure the proper administration of the drug through either route. This system also helped build trust and reduce the dropout rates of the participants.

A few drawbacks of this study need acknowledgement. Although both routes resulted in normalization of B12 levels, a total of 14/73 (19.2%) children remained anemic at the end of our study period. This was probably because most of these children had lower Hb levels to begin with and hence needed a duration longer than three months to achieve a nonanemic status.

In a prospective single-arm study conducted by Saxena et al., although normalization of B12 levels was achieved in all subjects, 67.6% of participants continued to remain anemic at six weeks. This was much higher than the value observed by us at 12 weeks duration, probably because our time point of assessment was much later [5].

Newer studies suggest that functional biomarkers like the holo-transcobalamin assay [17] may provide a more sensitive reflection of B12 activity than B12 levels both at baseline and follow-up. Also, elevated methylmalonyl-CoA and homocysteine levels act as important parameters to not only diagnose B12 deficiency but also help in differentiating it from folate deficiency [18]. The utility of these parameters is still not completely studied and may be an interesting area for future research.

Although we tried to objectively assess the compliance with therapy by checking the empty blister packs, we primarily relied on caregiver reporting to assess the method of administration. This could also be one of the reasons for the persistence of anemia in some patients. Moreover, factors like variations in dietary intake and intercurrent infections during the study period, which potentially could have hindered the outcomes, could not be taken into account.

Being a single-center study with a relatively small sample size, the generalizability of the findings to other populations is limited. The follow-up duration of three months was relatively short and did not allow assessment of long-term hematological response or relapse rates. Future multicentric studies with larger sample sizes, longer follow-ups, and incorporation of functional biomarkers are warranted to strengthen the current evidence.

## Conclusions

This study proves that the sublingual route is as effective as the IM route with respect to improvement of serum B12 levels. Although in cases with neurological involvement and pancytopenia the parenteral route may be preferred at the time of initiation of therapy, switching over to the sublingual route for maintenance may help improve compliance rates. Although multicentric studies with larger sample sizes and longer follow-up periods are needed to incorporate this route into guidelines, this study highlights that sublingual B12 may be considered as a potential option, pending evidence.
